# Flexitarian dietary patterns and neuropsychiatric multimorbidity among the oldest-old in China

**DOI:** 10.3389/fnut.2026.1789764

**Published:** 2026-05-11

**Authors:** Xixing Xu, Afei Qin, Shasha Wang

**Affiliations:** 1 Shandong Cancer Hospital and Institute, Shandong First Medical University and Shandong Academy of Medical Sciences, Jinan, China; 2 Department of Social Medicine and Health Management, School of Public Health, Cheeloo College of Medicine, Shandong University, Jinan, China; 3 NHC Key Lab of Health Economics and Policy Research, Shandong University, Jinan, China; 4 Center for Health Management and Policy Research, Shandong University (Shandong Provincial Key New Think Tank), Jinan, China; 5 School (Institute) of Mental Health and Psychological Sciences, Cheeloo College of Medicine, Shandong University, Jinan, China

**Keywords:** cognitive impairment, depression, flexitarian diet, nutritional epidemiology, the oldest-old

## Abstract

**Background:**

Flexitarian dietary patterns, characterized by diets mainly based on plant foods with limited intake of animal-source foods, have attracted growing interest as a sustainable and potentially healthy eating approach. However, evidence on their associations with mental and cognitive health in the oldest-old remains limited. This study aimed to investigate the associations between flexitarian dietary patterns and neuropsychiatric outcomes among older Chinese adults.

**Methods:**

Data were derived from 11,437 community-dwelling Chinese adults aged 65 years and older participating in the 2018 Chinese Longitudinal Healthy Longevity Survey. Dietary intake was assessed using a validated food frequency questionnaire, and participants were classified as omnivores or flexitarians, with flexitarians further categorized into pesco-flexitarian, ovo-lacto-flexitarian, and vegan-flexitarian subtypes. The co-occurrence of depression and cognitive impairment was defined as neuropsychiatric multimorbidity.

**Results:**

After multiple imputation and adjustment for a wide range of sociodemographic, health-related, and lifestyle factors, flexitarian diets were associated with higher odds of neuropsychiatric multimorbidity compared with omnivorous diets (OR 1.42, 95% CI 1.16–1.74). Higher odds were mainly observed among pesco-flexitarians (OR 1.92, 95% CI 1.28–2.89) and vegan-flexitarians (OR 1.52, 95% CI 1.10–2.09). Flexitarian dietary patterns were also linked to increased odds of depression (OR 1.20, 95% CI 1.09–1.33) and cognitive impairment (OR 1.29, 95% CI 1.10–1.50) when examined separately, with the strongest associations seen in vegan-flexitarians. Interaction analyses indicated that the association between flexitarian diets and neuropsychiatric multimorbidity was stronger in men (*p* = 0.006). These findings were consistent across multiple sensitivity analyses.

**Conclusion:**

In this nationally representative sample of older Chinese adults, flexitarian dietary patterns were not associated with better neuropsychiatric health and were instead linked to higher odds of depression, cognitive impairment, and their co-occurrence. These results suggest that plant-forward diets in the oldest-old should place greater emphasis on overall dietary quality and nutrient adequacy.

## 1 Introduction

Dietary patterns have increasingly been recognized as an important and modifiable determinant of health in older adults, with growing interest in plant-forward eating approaches for promoting healthy aging. A growing body of research has linked specific dietary components—such as mushrooms, garlic, vegetables, and fruits—to a reduced odds of depression and cognitive impairment (CI) (1, 2). In addition, several dietary patterns rich in plant-based foods, including the Mediterranean diet and the MIND diet, have demonstrated potential neuroprotective effects in older populations (3–6), supporting the hypothesis that plant-forward eating patterns may contribute to better neuropsychiatric health. However, these dietary patterns are typically characterized not by the exclusion of animal-source foods, but by their balanced integration within nutritionally diverse diets.

Against this background, flexitarian diets emphasize plant-based foods, including abundant intake of fruits, vegetables, whole grains, legumes, and nuts. At the same time, they allow moderate consumption of animal products, such as limited red meat and moderate intake of poultry, fish, eggs, and dairy, and have attracted increasing attention in recent years because of their flexibility, sustainability, and perceived feasibility for long-term adherence (7–9). Despite their growing popularity, large-scale population-based epidemiological evidence regarding the association between flexitarian diets and late-life neuropsychiatric outcomes remains scarce. Importantly, accumulating evidence indicates that the neuropsychiatric health effects of plant-forward or flexitarian diets are not uniform across populations, but may vary according to overall dietary quality, nutrient adequacy, and baseline nutritional status, particularly in older adults (10–15). In older populations with limited dietary diversity or already low intake of animal-source foods, further restriction of such foods may have unintended health consequences. This contextual variability underscores the need for population-specific evidence to inform dietary recommendations related to neuropsychiatric health in later life.

Depression and CI are common neuropsychiatric disorders that pose a substantial threat to the health of older adults, characterized by high prevalence and profound adverse outcomes. Globally, the prevalence of depression among older adults is estimated at 35.1% (95% CI 30.2–40.4) (16), and that of CI at 23.7% (95% CI 18.6–29.6) (17). In China, the prevalence of depression in older adults has been reported at 26.4% (18), affecting more than 80 million people, and the country has the largest number of individuals with dementia worldwide, a figure projected to rise further (19). Of particular concern is the frequent co-occurrence of depression and CI—termed neuropsychiatric multimorbidity (NPM) (20)—due to their bidirectional influence and overlapping pathological mechanisms (21–24). Evidence suggests that older adults with NPM, compared with those with either condition alone, experience accelerated functional decline, a heightened odds of dementia (25), markedly reduced quality of life, and a several-fold increase in mortality odds, presenting a major challenge to aging and care systems (25–28).

Given the limitations of pharmacological interventions in older populations—such as age-related changes in susceptibility to adverse drug reactions (29), polypharmacy, and drug–drug interactions (30)—and the difficulty in reversing disease progression (31), lifestyle-based strategies have become increasingly important for the prevention and management of NPM. Diet, as a lifelong and highly modifiable lifestyle factor, offers considerable potential for optimizing neuropsychiatric health (32, 33). However, most dietary evidence to date has primarily focused on single neuropsychiatric conditions rather than their co-occurrence, and has largely been derived from populations with relatively adequate nutritional resources, leaving uncertainty regarding the applicability of such findings to the oldest-old with complex multimorbidity and constrained dietary diversity.

To address this evidence gap and to better understand the context-specific health implications of flexitarian diets, we used data from the Chinese Longitudinal Healthy Longevity Survey (CLHLS), a large, nationally representative cohort of the oldest-old, to examine the association between flexitarian dietary patterns and the odds of neuropsychiatric multimorbidity (NPM) among community-dwelling older Chinese adults. Beyond the overall flexitarian pattern, we further assessed the independent associations of different flexitarian subtypes—pesco-flexitarian, ovo-lacto-flexitarian, and vegan-flexitarian—with NPM, depression, and cognitive impairment. By generating population-based evidence from a setting characterized by advanced age and relatively limited dietary diversity, this study seeks to inform more nuanced and context-sensitive dietary strategies for the prevention and management of NPM in the oldest-old.

## 2 Methods

### 2.1 Study population

Data were drawn from the CLHLS (34), one of the largest and longest-running prospective cohort studies worldwide with a focus on the oldest-old population in China. Notably, the CLHLS sample is characterized by a high proportion of very old adults, with a substantial share of participants aged 80 years and older. The CLHLS aims to investigate biological, social, behavioral, and environmental factors associated with healthy aging and longevity in older Chinese adults. To date, eight survey waves have been conducted (1998, 2000, 2002, 2005, 2008, 2011–2012, 2014, and 2018), providing a unique resource for studying the health of the oldest-old in China. In 2018, the survey covered 15,874 older adults from 23 provinces, autonomous regions, and municipalities in China. All participants were interviewed face-to-face by trained interviewers, with rigorous quality control procedures in place. Further details of the CLHLS design have been published elsewhere (35, 36).

**Figure 1:**
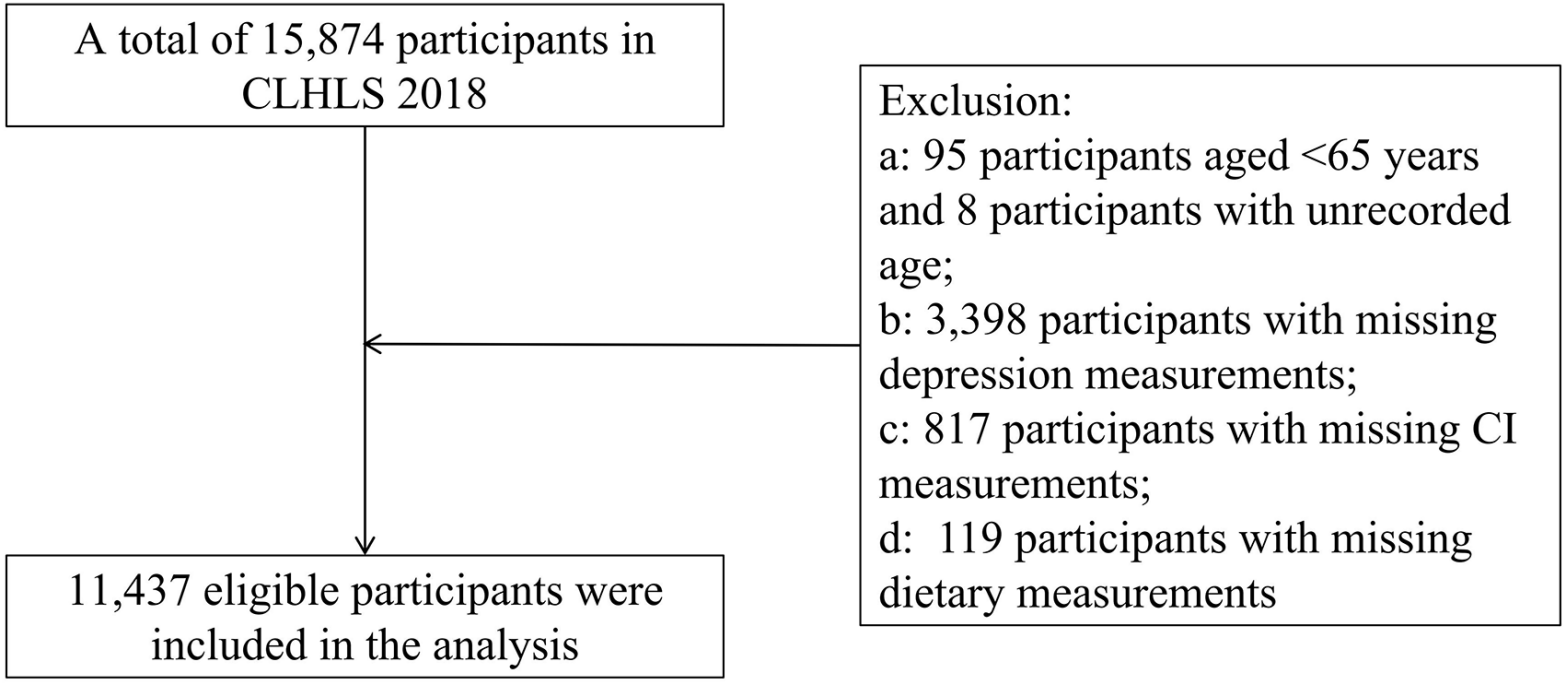
Sample screening process.

This study used cross-sectional data from the 2018 wave. Based on the study objectives, we excluded participants who: (1) were younger than 65 years; (2) lacked assessment of depression; (3) lacked assessment of cognitive function; or (4) lacked dietary information. A total of 11,437 older adults were included in the final analyses. The sample selection process is shown in Figure 1.

This study was approved by the biomedical ethics committee of Peking University (IRB00001052-13074). All participants or their legal representatives provided written informed consent for participation. Consent was obtained prior to the administration of the study questionnaire.

### 2.2 Institutional review board statement

This study was conducted according to the guidelines laid down in the Declaration of Helsinki, and all procedures involving research study participants were approved by the biomedical ethics committee of Peking University (IRB00001052-13074). All participants or their guardians provided written informed consent for participation. Consent was obtained prior to the administration of the study questionnaire.

### 2.3 Assessment of flexitarian diet

Dietary intake frequency was assessed using a simplified qualitative food frequency questionnaire (FFQ) designed according to international standards and tailored to Chinese dietary habits, with established validity and reliability in Chinese populations (37–40). The FFQ included 13 food categories: fruits, vegetables, meat, fish, eggs, legumes, salted vegetables, sugar, tea, garlic, milk or dairy products, nuts or nut products, and mushrooms or algae. For each food, participants reported intake frequency as: “almost every day”, “at least once per week but not daily”, “at least once per month but not weekly”, “occasionally but not monthly”, or “rarely or never”. To minimize potential recall bias among older adults, the CLHLS allowed the use of proxy respondents when participants were unable to complete the interview themselves. In such cases, dietary information was provided by close family members (e.g., spouse or adult children) who were familiar with the participants’ daily eating habits, a practice commonly adopted in studies of the oldest-old to improve data completeness and reliability.

In line with widely used definitions (41, 42), vegetarian status was determined based on the intake of four animal-based food groups: meat, fish, eggs, and dairy products. Following previous literature (7–9), flexitarian diets were defined as plant-based dietary patterns supplemented with limited amounts of animal-based foods—specifically, low red meat intake (about once per week), moderate intake of other animal-derived foods (poultry, fish, eggs, and dairy), and abundant consumption of plant-based foods (8). In this study, participants who reported eating meat “at least once per month but not weekly” or less were considered to consume small amounts of red meat. Accordingly, participants were categorized into omnivores (meat ≥ once per week) and flexitarians (meat ≤ three times per month). Flexitarians were further classified into: pesco-flexitarian: meat ≤ three times per month and fish ≥ once per week; ovo-lacto-flexitarian: meat ≤ three times per month and eggs or dairy ≥ once per week; vegan-flexitarian: meat, fish, eggs, and dairy all ≤ three times per month.

### 2.4 Assessment of NPM

NPM in this study comprised co-occurring depression and CI.

Depression was assessed using the 10-item Center for Epidemiologic Studies Depression Scale (CESD-10) (43). Each item was scored from 0 to 3 (rarely or none of the time to most or all of the time). Three reverse-coded items were recoded before summing the total score (range 0–30), with higher scores indicating more severe depressive symptoms. A score of ≥10 was used to define depression (44–46).

CI was assessed with the Chinese version of the Mini-Mental State Examination (MMSE), which consists of 24 items with a total score of 30; higher scores indicate better cognitive function. In line with previous studies, an MMSE score <18 was classified as severe CI (36, 47, 48). The details and validation of the Chinese MMSE have been established in earlier research (36). Following earlier definition (20), NPM was defined as meeting criteria for both depression and CI.

### 2.5 Assessment of covariates

Given the sociodemographic variation and known odds factors for neuropsychiatric conditions, we adjusted for a comprehensive set of covariates, including sociodemographic factors, economic factors, physical health, and lifestyle behaviors, consistent with previous studies (47, 49).

Sociodemographic factors included sex (male/female), residence (urban/town/rural), years of education (none, 1–6 years, >6 years), living arrangement (with family/alone), and marital status (married and living together/other: divorced, widowed, never married, married but living apart). Economic factors included timely access to medical services, pension, and health insurance (all coded yes/no).

Physical health factors included disability and history of chronic diseases. Disability was assessed using the Activities of Daily Living (ADL) scale; participants requiring assistance with any of six basic activities (bathing, dressing, toileting, indoor mobility, continence, and eating) were classified as disabled. Chronic disease history covered 13 conditions (e.g., diabetes, hypertension, heart disease) (47), categorized as none, one, or multimorbidity. Lifestyle behaviors included physical activity, smoking, and alcohol consumption (all coded yes/no). Physical activity was defined as having ever exercised or currently exercising; smoking and alcohol variables were coded similarly based on ever-use or current use.

### 2.6 Statistical analysis

All analyses were conducted using Stata MP version 17. Missing values were imputed for covariates only using multiple imputation by chained equations (mi impute chained), generating 20 imputed datasets with 20 iterations each. The proportions of missing data for all covariates included in the adjusted analyses are presented in Supplementary Table S1. Within the imputation models, binary covariates were imputed using logistic regression and unordered categorical covariates were imputed using multinomial logistic regression. The outcome variable, NPM, was not imputed but was included in the imputation model as an auxiliary variable to improve prediction of missing covariates. Estimates from the imputed datasets were combined using Rubin’s rules. For transparency, we additionally compared the observed prevalence of NPM between participants with complete covariate data and those requiring imputation for at least one covariate (Supplementary Table S2), and further examined NPM prevalence according to variable-specific missingness of covariates (Supplementary Table S3).

Firstly, descriptive and univariate analyses: Distributions of NPM and all categorical covariates were summarized as frequencies (*n*) and percentages (%). Differences across NPM status were assessed using χ^2^ tests combined across the 20 imputed datasets (mi estimate: svy: tab). Secondly, multivariable logistic regression: NPM (yes/no) was the dependent variable. First, a binary dietary pattern variable (omnivores vs. flexitarians) was entered as the main exposure; then, a four-category variable (omnivores, pesco-flexitarian, ovo-lacto-flexitarian, vegan-flexitarian) was analyzed. Fully adjusted odds ratios (OR) and 95% confidence intervals (95% CIs) were estimated. This analytical framework was also applied separately to depression and CI outcomes. Thirdly, in adjusted models for NPM, interaction terms between dietary pattern (binary) and age, sex, and residence were included to assess effect modification. All regressions were conducted with “mi estimate, or: logit”, pooling estimates across 20 imputed datasets. Logistic models were fitted on the log-odds scale, and the reported ORs and 95% CIs were obtained by exponentiating the regression coefficients and their corresponding confidence limits.

Three sensitivity analyses were performed: (1) Redefining the flexitarian group by excluding pesco-flexitarians [given that fish-containing vegetarian diets are sometimes not classified as vegetarian (50)]; (2) Repeating analyses in a complete-case dataset; and (3) Revising the MMSE cut-off for CI by education level (<18 for no schooling; <21 for 1–6 years; <25 for >6 years of education) and re-running the CI models. A two-sided *α* of 0.05 was considered statistically significant in all analyses.

## 3 Results

### 3.1 Study population characteristics

This study included 11,437 participants aged 65 years and older. Based on observed outcome data, the overall prevalence of NPM was 4.6% (526/11,437). The prevalence of NPM was lower among omnivores (4.0%) than among flexitarians (6.8%) (*p* < 0.001). Within the flexitarian subgroups, pesco-flexitarians (7.3%), ovo-lacto-flexitarians (6.4%), and vegan-flexitarians (7.2%) all had higher prevalences of NPM than omnivores (*p* < 0.001). Age, sex, residence, years of schooling, marital status, ability to get to hospital when ill, pension, medical insurance, disability status, exercise, smoking status, drinking status, history of disease, and dietary pattern were all significantly associated with NPM (all *p* < 0.05). The detailed distribution of participant characteristics according to NPM status is presented in Table 1.

**Table 1:** The baseline characteristics of older respondents with and without NPM.

Variables	Category	No NPM	NPM	Total	*χ^2^*	*P-*value
		*N* (%^a^)	*N* (%^a^)	*N* (%^a^)		
Total		10,911 (95.4)	526 (4.6)	11,437 (100)		
Age	65 to 74 years	2,869 (99.7)	8 (0.3)	2,877 (25.2)	491.167	<0.001
	75 to 84 years	3,490 (98.8)	41 (1.2)	3,531 (30.9)		
	≥85 years	4,552 (90.5)	477 (9.5)	5,029 (44.0)		
Sex	Men	5,159 (97.2)	150 (2.8)	5,309 (46.4)	71.048	<0.001
	Women	5,752 (93.9)	376 (6.1)	6,128 (53.6)		
Residence	Urban	2,668 (96.9)	85 (3.1)	2,753 (24.1)	18.885	<0.001
	Township	3,540 (94.9)	190 (5.1)	3,730 (32.6)		
	Rural	4,703 (94.9)	251 (5.1)	4,954 (43.3)		
Living arrangement	With family member	8,726 (95.6)	405 (4.4)	9,131 (79.8)	2.765	0.096
	Lives alone	2,185 (94.8)	121 (5.2)	2,306 (20.2)		
Years of schooling	Never	4,677 (91.7)	424 (8.3)	5,101 (44.6)	292.128	<0.001
	1 to 6 years	3,864 (98.0)	77 (2.0)	3,941 (34.5)		
	>6 years	2,370 (99.0)	25 (1.0)	2,395 (20.9)		
Marital status	Currently married and living together	5,043 (98.5)	77 (1.5)	5,120 (44.8)	202.406	<0.001
	Divorced, widowed, never married, or married but not living together	5,868 (92.9)	449 (7.1)	6,317 (55.2)		
Able to get to hospital when ill	Yes	10,658 (95.6)	490 (4.4)	11,148 (97.5)	41.723	<0.001
	No	253 (87.5)	36 (12.5)	289 (2.5)		
Pension	No	5,216 (94.4)	307 (5.6)	5,523 (48.3)	22.41	<0.001
	Yes	5,695 (96.3)	219 (3.7)	5,914 (51.7)		
Medical insurance	No	1,237 (94.1)	78 (5.9)	1,315 (11.5)	6.012	0.014
	Yes	9,674 (95.6)	448 (4.4)	10,122 (88.5)		
Disability status	No	9,075 (97.6)	224 (2.4)	9,299 (81.3)	543.877	<0.001
	Yes	1836 (85.9)	302 (14.1)	2,138 (18.7)		
Exercise	Never	6,235 (94.0)	400 (6.0)	6,635 (58)	73.602	<0.001
	Former or current	4,676 (97.4)	126 (2.6)	4,802 (42)		
Smoking status	Never	7,441 (94.5)	430 (5.5)	7,871 (68.8)	42.948	<0.001
	Former or current	3,470 (97.3)	96 (2.7)	3,566 (31.2)		
Drinking status	Never	7,886 (94.6)	449 (5.4)	8,335 (72.9)	43.471	<0.001
	Former or current	3,025 (97.5)	77 (2.5)	3,102 (27.1)		
History of disease	None	3,747 (94.6)	213 (5.4)	3,960 (34.6)	8.59	0.014
	One	3,346 (95.9)	142 (4.1)	3,488 (30.5)		
	Comorbidity	3,818 (95.7)	171 (4.3)	3,989 (34.9)		
Dietary pattern	Omnivores	8,632 (96.0)	359 (4.0)	8,991 (78.6)	36.268	<0.001
	Pesco-flexitarian	404 (92.7)	32 (7.3)	436 (3.8)		
	Ovo-lacto-flexitarian	1,183 (93.6)	81 (6.4)	1,264 (11.1)		
	Vegan-flexitarian	692 (92.8)	54 (7.2)	746 (6.5)		
Dietary pattern	Omnivores	8,632 (96.0)	359 (4.0)	8,991 (78.6)	35.213	<0.001
	Flexitarian	2,279 (93.2)	167 (6.8)	2,446 (21.4)		

NPM, neuropsychiatric multimorbidity.

a Due to rounding, the sum of percentages for some variable categories may not equal exactly 100%.

The prevalence of NPM among the oldest-old (≥85 years) was 9.5%, which was significantly higher than that in the 75–84 years group (1.2%) and the 65–74 years group (0.3%) (*p* < 0.001). Women had a higher prevalence of NPM than men (6.1% vs. 2.8%, *p* < 0.001). The prevalence among participants with no formal education was substantially higher than among those with more than 6 years of schooling (8.3% vs. 1.0%, *p* < 0.001). Individuals with disabilities also had a markedly higher prevalence of NPM than those without disabilities (14.1% vs. 2.4%, *p* < 0.001).

The proportions of missing data for covariates included in the adjusted analyses are presented in Supplementary Table S1. Participants requiring covariate imputation had a slightly higher observed prevalence of NPM than those with complete covariate data (5.1% vs. 4.3%), although the difference was not statistically significant (*p* = 0.075; Supplementary Table S2). Variable-specific comparisons of NPM prevalence according to covariate missingness are shown in Supplementary Table S3.

### 3.2 Association between flexitarian diets and NPM, depression, and CI

Multivariable-adjusted logistic regression analyses (Table 2) indicated that adherence to a flexitarian dietary pattern was significantly associated with higher odds of NPM (OR = 1.42, 95% CI: 1.16–1.74, *p* = 0.001). In subgroup analyses, pesco-flexitarians (OR = 1.92, 95% CI: 1.28–2.89, *p* = 0.002) and vegan-flexitarians (OR = 1.52, 95% CI: 1.10–2.09, *p* = 0.011) had significantly higher NPM odds compared with omnivores, whereas ovo-lacto-flexitarians did not reach statistical significance (OR = 1.23, 95% CI: 0.95–1.61, *p* = 0.121). For depression, flexitarian diets overall were associated with 20% higher odds (OR = 1.20, 95% CI: 1.09–1.33, *p* < 0.001), with the highest odds observed in vegan-flexitarians (OR = 1.46, 95% CI: 1.24–1.71, *p* < 0.001). Analyses of CI further corroborated the dietary pattern effect: overall, flexitarian diets were associated with a 29% higher odds (OR = 1.29, 95% CI: 1.10–1.50, *p* = 0.001). Both ovo-lacto-flexitarians (OR = 1.23, 95% CI: 1.01–1.50, *p* = 0.041) and vegan-flexitarians (OR = 1.42, 95% CI: 1.11–1.81, *p* = 0.005) had significantly higher odds of CI compared with omnivore controls.

**Table 2:** Associations between flexitarian dietary patterns and NPM, depression, and CI.

Items	NPM		Depression		CI	
	OR (95% CI)	*P* value	OR (95% CI)	*P* value	OR (95% CI)	*P* value
Model 1						
*Dietary pattern*						
Omnivores	1 (Reference)		1 (Reference)		1 (Reference)	
Flexitarian	1.42 (1.16, 1.74)	0.001	1.20 (1.09, 1.33)	<0.001	1.29 (1.10, 1.50)	0.001
Model 2						
Omnivores	1 (Reference)		1 (Reference)		1 (Reference)	
Pesco-flexitarian	1.92 (1.28, 2.89)	0.002	1.10 (0.88, 1.37)	0.395	1.24 (0.88, 1.74)	0.222
Ovo-lacto-flexitarian	1.23 (0.95, 1.61)	0.121	1.11 (0.97, 1.26)	0.140	1.23 (1.01, 1.50)	0.041
Vegan-flexitarian	1.52 (1.10, 2.09)	0.011	1.46 (1.24, 1.71)	<0.001	1.42 (1.11, 1.81)	0.005

NPM, neuropsychiatric multimorbidity; CI, cognitive impairment; OR, odds ratios; 95% CI, 95% confidence interval.

Both Model 1 and Model 2 were adjusted for age, sex, residence, living arrangement, years of schooling, marital status, ability to get to a hospital when ill, pension, medical insurance, disability status, exercise, smoking status, drinking status, and history of disease.

### 3.3 Interaction of flexitarian diet with age, sex, and residence in relation to NPM

Interaction analyses between dietary patterns and demographic variables showed no significant interactions between flexitarian diets and age groups (all *p* > 0.05), indicating that the NPM odds associated with flexitarian diets did not differ significantly across age categories. The interaction term for Flexitarian×Male was significant (OR = 1.84, 95% CI: 1.19–2.84, *p* = 0.006), suggesting that the association between flexitarian diets and NPM odds was markedly stronger among older men than among older women. No significant interactions were observed between flexitarian diets and place of residence (*p* > 0.05 for all), indicating that the flexitarian–NPM association was consistent across different residential settings (Table 3).

**Table 3:** Interaction effects of flexitarian dietary patterns with age, sex, and residence on the odds of NPM.

Items	OR (95% CI)	*P*
Flexitarian × Age^a^		
*Dietary pattern*		
Omnivores	1 (Reference)	
Flexitarian	1.93 (0.46, 8.13)	0.369
*Age group*		
65 to 74 years	1 (Reference)	
75 to 84 years	3.34 (1.28, 8.70)	0.013
≥85 years	15.09 (6.09, 37.42)	<0.001
Flexitarian × 65 to 74 years	1 (Reference)	
Flexitarian × 75 to 84 years	0.61 (0.12, 3.00)	0.542
Flexitarian × ≥ 85 years	0.74 (0.17, 3.18)	0.690
Flexitarian × Sex^b^		
*Dietary pattern*		
Omnivores	1 (Reference)	
Flexitarian	1.19 (0.93, 1.52)	0.157
*Sex*		
Women	1 (Reference)	
Men	0.84 (0.63, 1.11)	0.223
Flexitarian × Women	1 (Reference)	
Flexitarian × Men	1.84 (1.19, 2.84)	0.006
Flexitarian × Residence^c^		
*Dietary pattern*		
Omnivores	1 (Reference)	
Flexitarian	1.05 (0.59, 1.88)	0.862
*Residence*		
Urban	1 (Reference)	
Township	1.58 (1.13, 2.21)	0.008
Rural	1.31 (0.93, 1.83)	0.118
Flexitarian × Urban	1 (Reference)	
Flexitarian × Township	1.05 (0.53, 2.06)	0.888
Flexitarian × Rural	1.73 (0.91, 3.30)	0.093

OR, odds ratios; 95% CI, 95% confidence interval.

a Adjusted for sex, residence, living arrangement, years of schooling, marital status, ability to get to a hospital when ill, pension, medical insurance, disability status, exercise, smoking status, drinking status, and history of disease.

b Adjusted for age, residence, living arrangement, years of schooling, marital status, ability to get to a hospital when ill, pension, medical insurance, disability status, exercise, smoking status, drinking status, and history of disease.

c Adjusted for age, sex, living arrangement, years of schooling, marital status, ability to get to a hospital when ill, pension, medical insurance, disability status, exercise, smoking status, drinking status, and history of disease.

### 3.4 Sensitivity analyses

**Figure 2:**
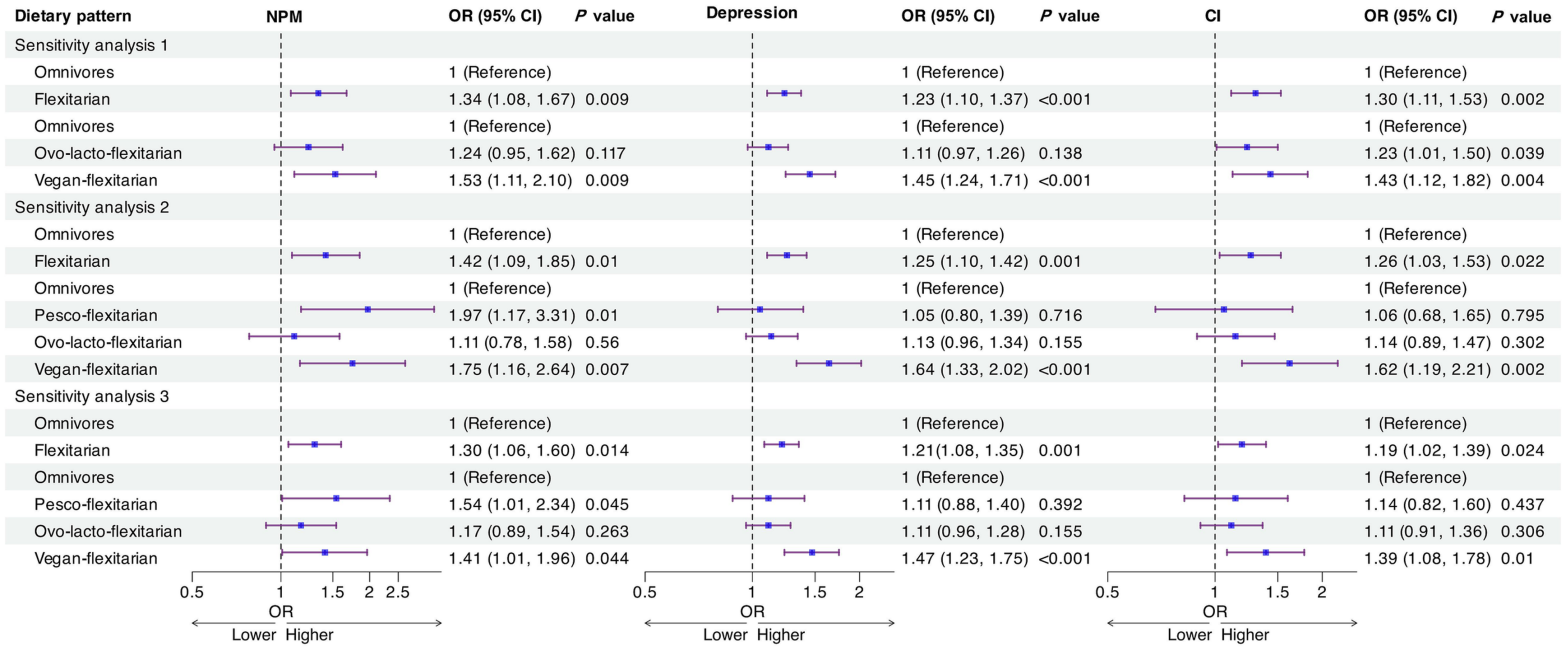
Sensitivity analyses of associations between dietary patterns and neuropsychiatric outcomes. NPM, neuropsychiatric multimorbidity; CI, cognitive impairment; OR, odds ratios; 95% CI, 95% confidence interval.

As shown in Figure 2, sensitivity analyses were consistent with the primary findings. After excluding pesco-flexitarians, adherence to a flexitarian diet remained significantly associated with higher odds of NPM (OR = 1.34, *p* = 0.009), depression (OR = 1.23, *p* < 0.001) and CI (OR = 1.30, *p* = 0.002). Among subgroups, vegan-flexitarians showed the largest odds increases (NPM: OR = 1.53; depression: OR = 1.45; CI: OR = 1.43; all *p* < 0.01).

In the complete-case analysis (excluding all participants with missing covariates), the associations persisted: flexitarian diets were associated with higher odds of NPM (OR = 1.42, *p* = 0.010), depression (OR = 1.25, *p* = 0.001) and CI (OR = 1.26, *p* = 0.022). Subgroup analyses again indicated increased odds among vegan-flexitarians (NPM: OR = 1.75; depression: OR = 1.64; CI: OR = 1.62; all *p* < 0.01). The association between ovo-lacto-flexitarian diets and CI was no longer significant (OR = 1.14, *p* = 0.302).

After applying education-specific MMSE cut-offs, flexitarian diets remained significantly associated with elevated odds of NPM (OR = 1.30, *p* = 0.014), depression (OR = 1.21, *p* = 0.001) and CI (OR = 1.19, *p* = 0.024). Subgroup analyses again showed higher odds for vegan-flexitarians (NPM: OR = 1.41; depression: OR = 1.47; CI: OR = 1.39; all *p* < 0.05), whereas the ovo-lacto-flexitarian—CI association remained non-significant (OR = 1.11, *p* = 0.306).

Three sensitivity analyses were conducted: Sensitivity analysis 1: Flexitarian group redefined by excluding pesco-flexitarians (based on classification standards where fish-containing diets are not considered vegetarian); Sensitivity analysis 2: Complete-case analysis using only participants with full covariate data; Sensitivity analysis 3: Revised CI definition using education-specific MMSE cut-offs (<18 for no schooling; <21 for 1–6 years; <25 for >6 years of education).

All sensitivity analyses were adjusted for age, sex, residence, living arrangement, years of schooling, marital status, ability to get to a hospital when ill, pension, medical insurance, disability status, exercise, smoking status, drinking status, and history of disease.

## 4 Discussion

In this large-scale, cross-sectional study based on a nationally representative sample of the oldest-old, we examined the associations between flexitarian dietary patterns (including pesco-flexitarian, ovo-lacto-flexitarian, and vegan-flexitarian diets) and NPM, depression alone, and CI. We found that, compared with non-flexitarians, overall flexitarianism was significantly associated with higher odds of NPM, depression, and CI, with particularly elevated NPM odds observed among pesco-flexitarians and vegan-flexitarians. Furthermore, analyses of sex modification effects suggested that this adverse association was more pronounced in men.

Compared with previous studies, our findings showed both consistencies and discrepancies. On the one hand, numerous studies on plant-based or Mediterranean dietary patterns have reported that diets rich in fruits, vegetables, whole grains, nuts, and healthy fats are associated with lower odds of depression and CI (4, 5, 10, 14), which is in contrast to the odds-increasing trend observed in our study. This discrepancy may be partly attributable to differences in the definition of dietary patterns: prior plant-based diet indices typically assign positive weights to high-quality plant foods and do not completely exclude animal-based foods (10, 14), whereas the flexitarian patterns in our study placed greater emphasis on avoiding frequent consumption of certain or all animal-based foods, potentially leading to inadequate intake of key nutrients. Additionally, differences in study populations, dietary assessment tools, and the scope of covariate adjustment may also contribute to divergent results. On the other hand, some studies are consistent with our findings. For example, research has reported that vegetarians and pescatarians in older age have a higher odds of fractures, sarcopenia, and frailty (15, 41, 51, 52)—factors that are strongly linked to cognitive decline and depression; another intervention study found that meat-containing diets were superior to lacto-ovo-vegetarian diets in improving muscle mass in older adults (53), which aligns with the potential odds direction suggested in our study. Moreover, some studies have found higher odds of depression among vegetarians (11, 12), as well as increased odds of stroke and dementia in older vegetarians (13). Overall, the current evidence on the relationships between plant-based, vegetarian, or flexitarian diets and mental or cognitive health is inconsistent, with substantial variation in both effect direction and magnitude, suggesting considerable uncertainty in this field. Further high-quality longitudinal and interventional studies across diverse populations and dietary cultures are warranted to verify and explain causal relationships and potential mechanisms.

These results are noteworthy because they appear to contrast with much of the evidence from western populations, where plant-forward dietary patterns such as the Mediterranean diet or the MIND diet are often linked to lower odds of depression and dementia (54). Several explanations may account for this discrepancy. First, definitions of “flexitarian” vary substantially across cultural contexts. In China, flexitarianism in the oldest-old often arises not from conscious health-seeking choices, but rather from economic constraints, reduced appetite, dental problems, or traditional preferences for a grain- and vegetable-based diet with limited animal-source foods. National surveys have shown that dietary protein intake among Chinese elderly has steadily declined from the early 1990s to the present, with a growing proportion falling below recommended nutrient intakes (55). Moreover, insufficient consumption of animal-source foods is a major contributor to micronutrient deficiencies such as vitamin B12, iron, and zinc, which are highly prevalent among older Chinese with low dietary diversity (56). Evidence also suggests that inadequate animal protein intake may be linked to higher odds of depression and anxiety, whereas higher-quality protein from mixed or animal sources is associated with better psychological outcomes (12, 57). By contrast, western plant-forward dietary models, such as the Mediterranean or MIND diets, emphasize balance and diversity, typically incorporating fish, nuts, and dairy products to ensure adequate high-quality protein and omega-3 fatty acids. Moreover, the bioavailability of certain nutrients may differ between plant- and animal-derived foods. As a result, nutritional supplementation or plant-based substitution may not fully offset reduced intake of animal-source foods unless nutrient form, source, and absorptive efficiency are also taken into account, especially in the oldest-old. This cultural and nutritional divergence may partly explain why, in our study, flexitarian diets were associated with increased rather than decreased odds of late-life neuropsychiatric conditions.

From a nutritional and biological perspective, insufficient energy and protein intake may be a key explanation (15). Flexitarians, when reducing animal food consumption, often lower their intake of total energy, high-quality protein, long-chain n-3 fatty acids (EPA, DHA), and various micronutrients such as vitamin B12, iron, zinc, and vitamin D (58, 59). These nutrients are crucial for neurotransmitter synthesis (60), anti-inflammatory responses (61), and the maintenance of muscle mass and bone health (58, 62); their deficiency may concurrently increase the odds of both depression and CI. In particular, adequate DHA intake has been shown to slow the progression from normal cognition to dementia in APOE4 carriers (63). Although some studies have suggested that plant-rich diets may benefit neuropsychiatric health through antioxidant, anti-inflammatory, and gut microbiota–modulating pathways (64, 65), such benefits strongly depend on appropriate nutrient composition and sufficient protein/energy intake. Imbalanced nutrient composition (66, 67) and inadequate protein and energy intake (15) in plant-based diets—especially in older adults, whose anabolic efficiency for plant protein is lower (15)—may limit their health benefits. In our study, pure flexitarians exhibited higher odds for multiple outcomes, suggesting that in older populations, plant-forward diets lacking adequate supplementation or substitution of key nutrients may have adverse consequences. Moreover, age-related declines in digestive and absorptive capacity, coupled with monotonous dietary patterns, may further amplify the negative effects of nutrient deficiencies.

Non-nutritional explanations are also important and should not be overlooked. First, reverse causation is a major limitation of cross-sectional studies: the oldest-old may reduce meat consumption due to chronic diseases, decreased appetite, impaired chewing function, or medical advice, making low animal food intake more a marker of disease or functional limitation than its cause. Although our analyses adjusted for multiple health and sociodemographic covariates (e.g., disability, chronic diseases, socioeconomic indicators), residual confounding or unmeasured health selection effects (e.g., dental status, food accessibility, use of nutritional supplements, long-term weight change) may remain. Second, dietary exposure was assessed via a simplified food frequency questionnaire lacking quantitative information on food amounts, energy, and key nutrients, as well as biomarker validation (e.g., serum vitamin B_12_, vitamin D, red blood cell fatty acid profiles), which may have introduced misclassification and information bias. Furthermore, the composition of flexitarian diets can vary widely across individuals—for example, some may consume little meat but sufficient fish, eggs, and dairy, while others may have inadequate total energy or protein intake—leading to substantial heterogeneity in nutritional status within the same dietary label, which could influence both the direction and magnitude of associations.

Regarding sex differences, we found that the association between flexitarianism and NPM was stronger in men than in women. This suggests that the impact of flexitarian diets on neuropsychiatric health may not be uniform across populations and could be modified by sex. Such differences have implications for future research and public health practice. Epidemiological studies should routinely consider sex-stratified analyses and interaction testing to identify high-odds subgroups, and dietary guidance or interventions should incorporate sex as a potential modifying factor to achieve more precise nutrition and health management.

The major strengths of this study include the use of a large, nationally representative dataset from the CLHLS, standardized depression and cognitive function scales, multiple imputation for missing data, and extensive sensitivity analyses to test the robustness of results. We also conducted sub-type analyses of flexitarian diets (pesco-, ovo-lacto-, and vegan-flexitarianism), providing initial evidence on the potential differential effects of various animal-source food exclusions. Importantly, our analytical focus was not on depression or CI alone but on their co-occurrence (NPM), making the examination of diet–NPM associations more reflective of the real and complex health odds faced by the oldest-old, with greater public health relevance.

Several limitations should be acknowledged when interpreting our findings. First, the cross-sectional design precludes the establishment of temporal relationships or causal inference between flexitarian dietary patterns and NPM. Second, dietary exposure was assessed using a simplified qualitative food frequency questionnaire, without quantitative information on food amounts, total energy intake, or key nutrients, and without biomarker validation. This may have led to exposure misclassification and limited our ability to examine specific nutritional pathways underlying the observed associations. Third, selection bias cannot be entirely ruled out. The present analyses were based on community-dwelling older adults who completed dietary and neuropsychiatric assessments in the 2018 CLHLS wave. Given that the CLHLS predominantly includes very old individuals, with a substantial proportion aged 80 years and above, the analytical sample may represent a subgroup of the oldest-old who survived to advanced age and were sufficiently healthy to participate in the survey. As such, this population may differ systematically from the broader population of adults aged ≥65 years in China. Consequently, our findings may not be fully generalizable to younger-old adults or to older individuals with poorer health or functional status. Fourth, although we adjusted for a wide range of sociodemographic, health-related, and lifestyle covariates, residual confounding may persist, particularly from unmeasured factors such as dental status, long-term nutritional status, use of micronutrient supplementation, food accessibility, and psychosocial support. Fifth, the operational definition and classification thresholds for flexitarian diets involved a degree of subjectivity, which may limit comparability across studies using alternative dietary definitions or indices. Finally, the study population consisted exclusively of older Chinese adults, and caution is warranted when extrapolating the findings to populations with different cultural, dietary, or healthcare contexts. Future studies should prioritize longitudinal designs to clarify temporal relationships, incorporate quantitative dietary assessment and biomarker validation, and further explore the nutritional and biological mechanisms linking dietary patterns to NPM. Comparative studies across populations with diverse dietary structures will also be essential for informing context-sensitive dietary guidance for aging societies.

Based on these findings and limitations, we propose cautious recommendations for clinical and public health practice. Flexitarianism should not be assumed to be inherently beneficial or harmful; rather, emphasis should be placed on overall dietary quality and nutrient adequacy in the oldest-old. For those reducing animal-source food intake, attention should be paid to ensuring adequate intake of vitamin B12, vitamin D, long-chain *ω*-3 fatty acids, high-quality protein, iron, and zinc—either through nutrient-rich foods or, when necessary, supplementation. In clinical and primary care settings, dietary patterns, weight changes, appetite, and food accessibility should be routinely considered when assessing the mental and cognitive health of the oldest-old. For future research, priority should be given to prospective cohort studies to clarify temporal relationships; more precise dietary assessment (quantitative intake, cooking methods, food sources) combined with blood/metabolic biomarkers to verify nutritional mediation pathways; and, where possible, dietary intervention or quasi-experimental studies to test the effects of specific dietary modifications on depression and cognitive outcomes. Further exploration of sex, socioeconomic status, oral health, and chronic disease status as modifiers or mediators of diet–neuropsychiatric health relationships is also warranted.

## 5 Conclusion

In this nationally representative study of Chinese oldest-old, flexitarian dietary patterns were associated with higher odds of NPM, depression, and cognitive impairment compared with omnivorous diets. Although these associations were statistically significant, the observed effect sizes were modest and warrant cautious interpretation. Nevertheless, this study contributes novel evidence highlighting the need for caution when applying “one-size-fits-all” dietary guidance in the context of global aging, and underscores the importance of tailoring recommendations to population-specific nutritional and health profiles. Our findings emphasize the need to consider overall dietary quality and nutrient adequacy when promoting plant-forward diets among the oldest-old. Future longitudinal studies are needed to confirm these associations and clarify underlying mechanisms.

## Data Availability

The data analyzed in this study were obtained from the Chinese Longitudinal Healthy Longevity Survey (CLHLS), which is hosted on the Peking University Open Research Data Platform. Access to the dataset is subject to approval by the data provider and requires a formal application through the CLHLS data repository. The data are not publicly available without permission but can be accessed by qualified researchers upon application and approval from the data administrators. Requests to access the dataset should be directed to the CLHLS Data Management Team via https://opendata.pku.edu.cn/dataverse/CHADS.
