# Supplementary material for: Flexitarian dietary patterns and neuropsychiatric multimorbidity among the oldest-old in China
Source: Front Nutr. 2026 May 11;13:1789764. doi: 10.3389/fnut.2026.1789764 (PMC13200527; doi:10.3389/fnut.2026.1789764)
Supplement: Supplementary file 1 [file Table_1.pdf]

Supplementary appendix

Table S1 Missingness of covariates included in the adjusted analyses

Variable	Total N	Missing n	Missing %	Non-missing n	Non-missing %
Age	11437	0	0	11437	100
Sex	11437	0	0	11437	100
Years of schooling	11437	1630	14.25	9807	85.75
Residence	11437	0	0	11437	100
Living arrangement	11437	138	1.21	11299	98.79
Marital status	11437	104	0.91	11333	99.09
Able to get to hospital when ill	11437	107	0.94	11330	99.06
Pension	11437	627	5.48	10810	94.52
Medical insurance	11437	386	3.38	11051	96.62
Exercise	11437	160	1.4	11277	98.6
Smoking status	11437	164	1.43	11273	98.57
Drinking status	11437	196	1.71	11241	98.29
Disability status	11437	335	2.93	11102	97.07
History of disease	11437	1718	15.02	9719	84.98

Note: Missing n indicates the number of participants with missing data for each covariate, and Missing % indicates the corresponding percentage. Non-missing n indicates the number of participants with available data, and Non-missing % indicates the corresponding percentage.

Table S2. Comparison of NPM prevalence according to covariate imputation status

Group	N	NPM, n (%)	95% CI	P value
Full analytic sample	11,437	526 (4.6)	4.2–5.0	–
Complete covariate data	7,182	311 (4.3)	3.9–4.8	0.075
At least one covariate imputed	4,255	215 (5.1)	4.4–5.8	

Note: NPM, neuropsychiatric multimorbidity. “Complete covariate data” indicates participants with complete data for all covariates included in the multiple imputation procedure. “At least one covariate imputed” indicates participants with missing data in at least one of these covariates. The P value was derived from the two-sample test of proportions comparing NPM prevalence between the two covariate-imputation groups.

Table S3. NPM prevalence according to variable-specific missingness of covariates

Variable	Group	N	NPM, n (%)	95% CI	P value
Age	Non-missing	11,437	526 (4.6)	4.2–5.0	—
	Missing	0	0	–	
Sex	Non-missing	11,437	526 (4.6)	4.2–5.0	—
	Missing	0	0	–	
Years of schooling	Non-missing	9,807	457 (4.7)	4.2–5.1	0.446
	Missing	1,630	69 (4.2)	3.3–5.2	
Residence	Non-missing	11,437	526 (4.6)	4.2–5.0	—
	Missing	0	0	–	
Living arrangement	Non-missing	11,299	516 (4.6)	4.2–5.0	0.135
	Missing	138	10 (7.2)	2.9–11.6	
Marital status	Non-missing	11,333	519 (4.6)	4.2–5.0	0.297
	Missing	104	7 (6.7)	1.9–11.6	
Able to get to hospital when ill	Non-missing	11,330	525 (4.6)	4.2–5.0	0.069
	Missing	107	1 (0.9)	0.0–2.8	
Pension	Non-missing	10,810	481 (4.4)	4.1–4.8	0.002
	Missing	627	45 (7.2)	5.2–9.2	
Medical insurance	Non-missing	11,051	503 (4.6)	4.2–4.9	0.195
	Missing	386	23 (6.0)	3.6–8.3	
Exercise	Non-missing	11,277	521 (4.6)	4.2–5.0	0.37
	Missing	160	5 (3.1)	0.4–5.8	
Smoking status	Non-missing	11,273	521 (4.6)	4.2–5.0	0.34
	Missing	164	5 (3.0)	0.4–5.7	
Drinking status	Non-missing	11,241	517 (4.6)	4.2–5.0	0.996
	Missing	196	9 (4.6)	1.7–7.5	
Disability status	Non-missing	11,102	514 (4.6)	4.2–5.0	0.367
	Missing	335	12 (3.6)	1.6–5.6	
History of disease	Non-missing	9,719	410 (4.2)	3.8–4.6	<0.001
	Missing	1,718	116 (6.8)	5.6–7.9	

Note: NPM, neuropsychiatric multimorbidity. For each covariate, participants were grouped according to whether data for that variable were missing or non-missing. P values were derived from chi-square tests comparing NPM prevalence between missing and non-missing groups for each covariate. Variables with no missing data are shown for completeness.
